# Supplementary material for: Tobacco Smoke Exposure According to Location of Home Smoking in Israel: Findings from the Project Zero Exposure Study
Source: Int J Environ Res Public Health. 2023 Feb 16;20(4):3523. doi: 10.3390/ijerph20043523 (PMC9965201; doi:10.3390/ijerph20043523)
Supplement: Supplementary file 1 [file ijerph-20-03523-s001.zip › ijerph-2120372-supplementary.pdf]

Supplemental Table S1: Tobacco smoke exposure of children (yes/no, as assessed by hair nicotine) by socioeconomic and other variables (N=141, children with sufficient hair for analysis only)

		**Percent Unexposed (<LOD)**		**Percent Exposed (>LOD)**		**Total**		**p-value**
		n	%	n	%	n	%	
Location	Garden/Yard/Out of house	12	28.6	30	71.4	42	29.8	0.38
	Balcony only	21	38.2	34	61.8	55	39.0	
	Designated indoor area	11	26.8	30	73.2	41	29.1	
	Whole house	0	0.0	3	100.0	3	2.1	
Child sex	Female	24	32.0	51	68.0	75	53.2	0.83
	Male	20	30.3	46	69.7	66	46.8	
Smoking parent	Mother only	8	36.4	14	63.6	22	15.6	0.84
	Father only	16	29.6	38	70.4	54	38.3	
	Both mother and father	20	30.8	45	69.2	65	46.1	
Parents' nationality	Both Israeli born	30	31.9	64	68.1	94	66.7	0.80
	Other	14	29.8	33	70.2	47	33.3	

Monthly household income	Lower/Average	23	30.7	52	69.3	75	55.2	0.79
	Above average	20	32.8	41	67.2	61	44.8	
Father's education	Not academic	13	18.1	59	81.9	72	53.3	0.001
	Academic[a]	29	46.0	34	54.0	63	46.7	
Mother's education	Not academic	12	26.7	33	73.3	45	32.4	0.45
	Academic[a]	31	33.0	63	67.0	94	67.6	

[a]Academic: With at least some University education, versus without any University education

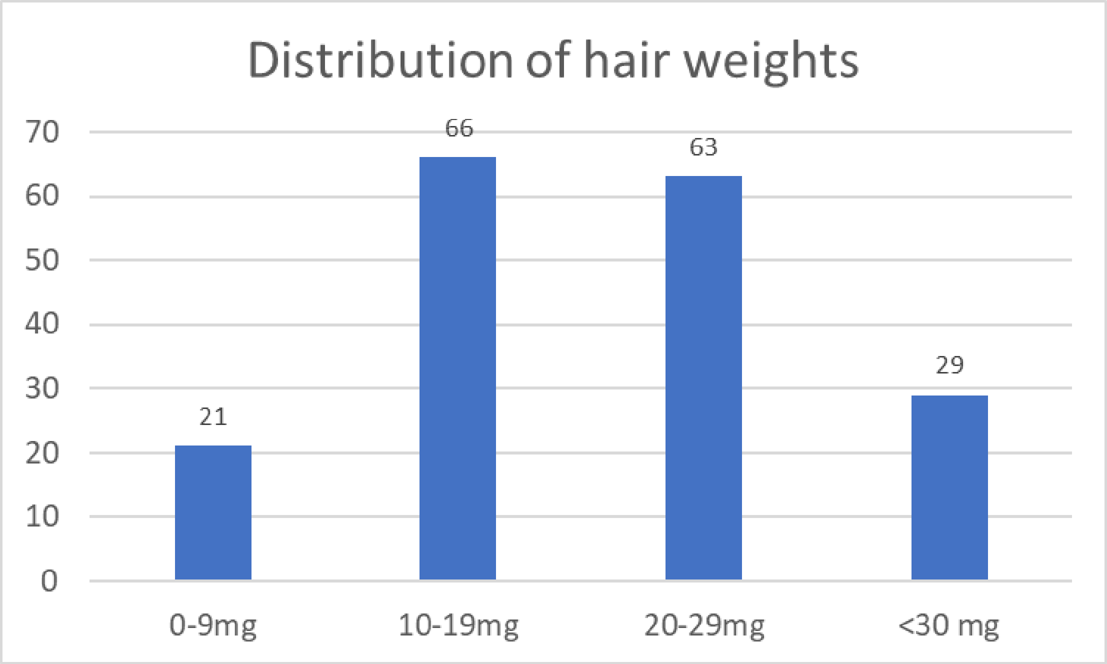

Supplemental Figure S1: Histogram of hair mass of participants in Studies 1 and 2.

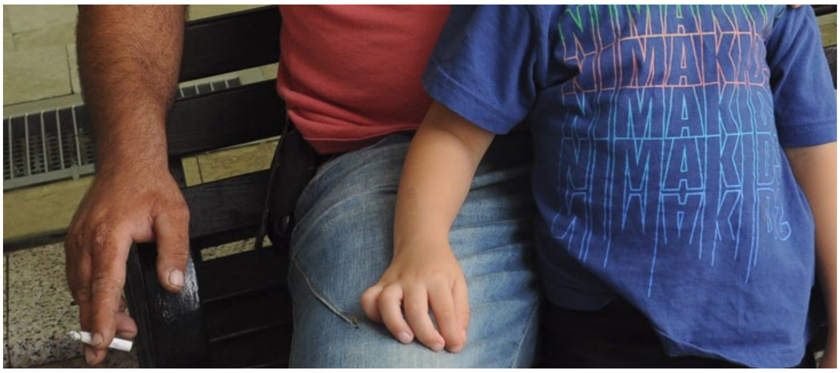

Supplemental Figure S2: Father smoking next to child. Jerusalem, outdoors.

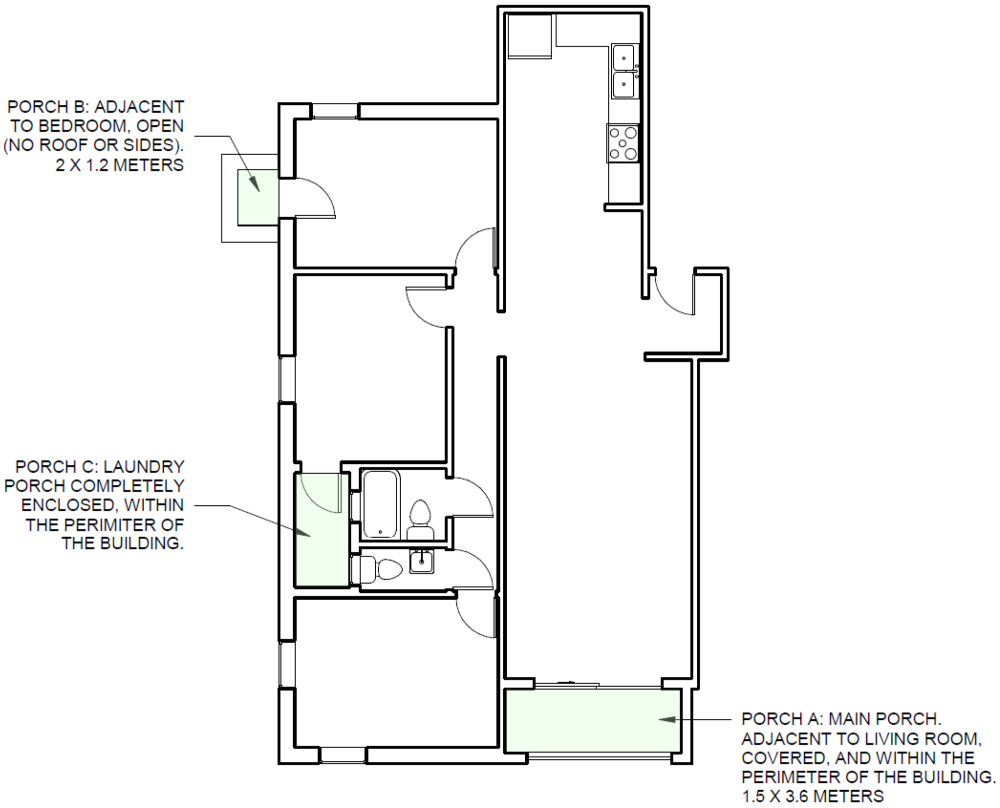

Supplemental Figure S3: Floor plan for an apartment with porches.

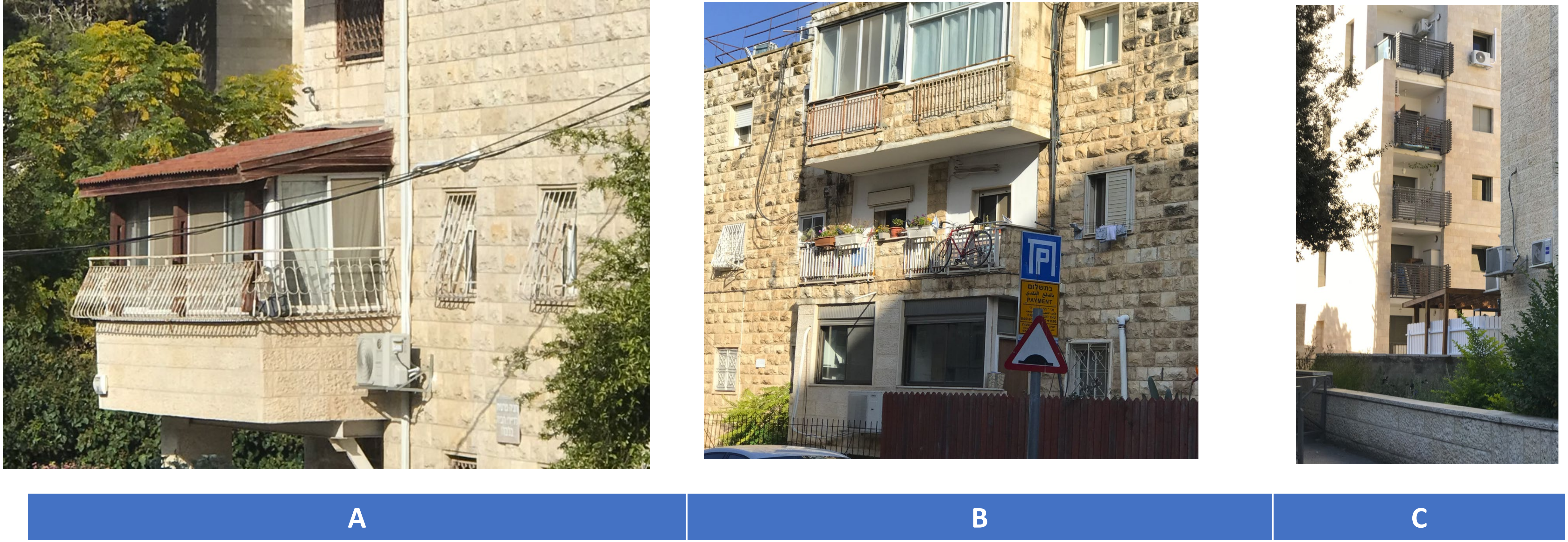

Supplemental Figure S4: Photos of typical porches in Jerusalem. From left to right: A. Porch outdoors, adjacent to the building, and completely enclosed. B. Top and bottom apartments have completely enclosed porches, while the middle apartments have covered, semi-enclosed porch. C. Porches stacked one on top of each other, all adjacent to the apartments, all except top apartment completely covered, all partially enclosed, all partially within the building perimeter, all small.
